# Shifts in primary care utilization patterns following digital health implementation: an operational evaluation of the 2023–2024 Hajj mass gatherings

**DOI:** 10.3389/fpubh.2026.1858949

**Published:** 2026-08-06

**Authors:** Amr A. Amin, Mohammad Althubiti, Ahmed A. Hawsawi, Mohammed S. Malki, Reem M. Allam, Neda M. Bogari

**Affiliations:** 1 Department of Biochemistry, Faculty of Medicine, Umm Al-Qura University, Makkah, Saudi Arabia; 2 King Faisal Hospital, Makkah, Saudi Arabia; 3 Department of Clinical Pathology, Faculty of Medicine, Zagazig University, Zagazig, Egypt; 4 Department of Genetics, Faculty of Medicine, Umm Al-Qura University, Makkah, Saudi Arabia

**Keywords:** digital triage, Hajj season, health care management risk, health equity, mass gathering medicine, surveillance systems

## Abstract

**Background:**

Early identification of care-seeking patterns and clinical triage optimization are critical challenges in mass gathering medicine. Genetic and structural clinical protocols are designed to manage surges, but the combined impact of digital triage networks on physical healthcare-seeking behaviors remains to be characterized. We evaluated the integration of a ‘Digital-First' healthcare model during the 2023–2024 Hajj mass gatherings, tracking shifts in primary care utilization patterns alongside extreme environmental thermal pressures.

**Methods:**

This prospective-observational cohort study utilized de-identified operational health registry data from the Saudi Ministry of Health (MOH) spanning the 2023 and 2024 Hajj seasons. High-fidelity clinical registry metrics from physical primary health care (PHC) clinics, emergency departments (ED), and specialized holy site hospitals were standardized as rates per 10,000 registered pilgrims. Digital care-seeking volumes were extracted from the unified Sehhaty virtual portal. A Sequential Explanatory Expert Forum comprising 15 senior critical care and public health specialists resolved operational recommendations.

**Results:**

The total registered pilgrim population was 1,845,223 in 2023 and 1,833,164 in 2024, representing a baseline density decrease of 0.65%. The implementation of the first digital model in 2024 was associated with the absorption of 516,585 virtual consultations, establishing a virtual-to-physical primary utilization ratio of 3.24:1. This digital deployment co-occurred with an 18.65 % decline in physical PHC visits (from 195,974 in 2023 to 159,425 in 2024), representing a standardized rate reduction from 1,062.1 to 869.7 visits per 10,000 pilgrims. Conversely, acute care strain rose sharply; holy site ED visits increased by 42.45% (from 212.0 to 304.0 per 10,000 pilgrims), while documented heat stroke cases rose by 73.36 % (from 6.1 to 10.6 per 10,000 pilgrims) and heat exhaustion rose by 42.10 % (57.2 to 81.8 per 10,000 pilgrims).

**Conclusions:**

The transition to a digital-first operational model co-occurred with a substantial shift in primary healthcare utilization, successfully redirecting low-acuity demand to virtual platforms during a mass gathering. However, this digital absorption did not decouple health system strain in acute care categories, where a documented environmental climate anomaly drove severe emergency and thermal illness surges. These findings suggest that virtual platforms function as critical clinical buffers rather than system-wide protectors during extreme climate events.

## 1 Introduction

Global health security is increasingly defined by how national infrastructures withstand the acute “pulse pressure” of Mass Gatherings (MGs). These events serve as rigorous stress tests for health systems, exposing latent vulnerabilities that remain invisible during routine operations (1). While historical MG medicine focused primarily on on-site trauma and infectious disease containment, the contemporary challenge has shifted toward systemic resilience—specifically, the ability to maintain standard-of-care delivery despite exponential surges in demand (2). Recent systematic reviews identify “Management Risks” rather than biological hazards as the primary point of failure; specifically, the operational silos and delayed risk assessments that prevent rapid adaptation to evolving threats (3–5).

The “Health Equity Imperative” further complicates this landscape. The sheer density of a mass gathering often creates a “dual burden” where the surge in low-acuity cases (heat exhaustion, minor injuries) overwhelms triage capacity, effectively blocking access for critical patients and disproportionately affecting vulnerable populations with pre-existing comorbidities (6). The traditional model of merely scaling up physical assets—more beds, more staff—is no longer fiscally or operationally sustainable (7).

The Hajj pilgrimage offers a unique epidemiological laboratory to validate new paradigms in crowd health. Unlike static events, the Hajj involves the synchronous, high-velocity movement of millions across distinct geographical nodes under extreme climatological conditions. The 2024 season, characterized by unprecedented thermal stress, provided a critical validation window for the Kingdom's “Digital-First” health transformation under Vision 2030 (8, 9).

This study provides a descriptive operational evaluation of how the implementation of integrated digital triaging and virtual medical networks correlates with primary health care utilization patterns during a mass gathering. By tracking the comparative distribution of low-acuity virtual consultations and physical clinic visits amid documented environmental pressures, we evaluate the capacity of digital health platforms to serve as operational buffers for physical healthcare infrastructure under seasonal surge conditions (10, 11).

## 2 Methodology

### 2.1 Operational dataset and consent

All clinical, demographic, and operational healthcare utilization datasets evaluated in this study were extracted directly from officially published, publicly accessible administrative health reports and annual statistical yearbooks compiled by the Ministry of Health (MOH), Saudi Arabia. Because the study relied entirely on pre-existing, aggregate-level, and fully anonymized public domain data, it did not involve direct patient contact, individual clinical records, or identifiable personal health information. Consequently, this investigation did not require formal institutional review board (IRB) review or specific ethical approval under national research regulations. For the qualitative component of this study, informed consent protocols were strictly maintained, with all 15 clinical and public health experts participating in the sequential explanatory forum (detailed in Section 2.4) providing written informed consent prior to their inclusion.

- **The Risk Domain Framework**: Operational performance was evaluated against the Management Risk domain specifically aiming to identify solutions for communication silos, lack of surveillance, and resource coordination failures.
- **The Health Equity Framework:** System outcomes were assessed for their ability to mitigate the “infrastructure constraints” that disproportionately affect vulnerable populations in resource-limited settings.

### 2.2 Quantitative analysis: operational stress markers

We analyzed aggregated quantitative operational data obtained from reports and institutional summaries, including aggregated laboratory testing volumes, service utilization indicators, and surveillance outputs recorded by the Saudi Ministry of Health (MOH) covering the 2023 and 2024 Hajj seasons (pilgrims *N* = 1.845 and 1.833 million, respectively). Data extraction focused on the “Operational Stress Markers” detailed in Table 1. The specific metrics selected for analysis included:

- Environmental Burden: Incidence rates of heat stroke and heat exhaustion, serving as proxies for external operational pressure.
- System Utilization: Comparative volume of physical Primary Health Care (PHC) visits versus virtual consultations (*Sehhaty* platform) to quantify the efficacy of the diversion strategy.
- Critical Care Capacity: Volume of emergency room (ER) visits, cardiac interventions, and renal dialysis sessions at Holy Sites hospitals.

**Table 1:** Operational stress markers and health system response matrix (Hajj Season 2023 vs. 2024).

Operational domain	Metric (Volume)	Season 2023 (*N* = 1,845,223)	Season 2024 (*N* = 1,833,164)	Volume change Δ%	Standardized Rate per 10,000 (2023)	Standardized Rate per 10,000 (2024)	Operational context & hypothesis-driven interpretation
1. Environmental burden							
*Climatological impact*	Heat stroke cases	1,120	**1,942**	**+73.4%**	**6.07**	**10.59**	**Supports Operational Rationale**: The sharp increase in thermal pathology supports the routing of acute heat illnesses to specialized Field Stabilization Units rather than immediate hospital transit, minimizing ER bottlenecking.
	Heat exhaustion cases	10,555	**14,997**	**+42.1%**	**57.20**	**81.81**	
2. The digital shift							
*Primary triage nodes*	**Physical** PHC Visits	**195,974**	**159,425**	**-18.6%**	**1,062.06**	**869.67**	**Aligns with Utilization Shifts**: The reduction in physical primary care foot traffic co-occurred with the absorption of over 516,000 virtual consultations, suggesting a shift in low-acuity healthcare-seeking behavior.
	**Virtual** consultations^*^	***–***	**516,585**	*Active*	**0.00**	**2,817.96**	
3. Acute care demand							
*Emergency pressure*	ER visits (Holy Sites)	39,118	55,717	+42.4%	**211.99**	**303.95**	**Reflects System Stress:** surge in ER volume highlights that acute, life-threatening cases bypassed primary care filters, justifying the preservation of physical hospital beds for critical triage.
*Critical interventions*	Cardiac -catheterization	1,261	1,270	+0.7%	**6.83**	**6.93**	**Sustained Capacity Hypothesis**: Suggests that while environmental stress increased, the baseline incidence of severe, acute ischemic coronary events requiring urgent intervention remained constant.
	Renal dialysis sessions	2,903	2,329	−19.8%	**15.73**	**12.70**	**Rescheduling Hypothesis**: Likely reflects improved pre-Hajj health screening regulations in source countries or streamlined patient scheduling via the Sehhaty platform.

^*^Virtual Consultations figure combines “Completed Virtual Appointments” (225,296) and “Instant Consultations” (291,289) from Makkah Health Cluster.

Pilgrim denominators used for rate calculations are 1,845,223 (2023) and 1,833,164 (2024), representing a density decrease of 0.65%. Comparative analysis illustrating the strategic decoupling of pilgrim density from physical health system strain. Despite a 73.4% escalation in environmental risk (Heat Stroke), physical primary care footfall declined by 18.6%, indicating the supportive efficacy of the Digital-diversion policy in preserving emergency capacity for acute surges (29).

Note on Data Provenance: To ensure full traceability, all operational data rows are mapped to their official, citable sources. Total registered pilgrim counts, physical PHC visits, Holy Site ER visits, Virtual consultation, Heat stroke and heat exhaustion numbers, open-heart procedures, cardiac catheterizations, and renal dialysis volumes are sourced from the Ministry of Health Annual Hajj Statistical Yearbook (2023 and 2024 editions) includes the records from the Sehhaty Platform National Operational Logs and National Health Command and Control Center (CCC) Thermal Injury Registry (Makkah Health Cluster), and the Centralized Laboratory Information Management System (LIMS) Season Reports.

These datasets were stratified to assess the system's “surge capacity” and to validate the hypothesis that digital diversion can effectively decouple rising crowd density from physical facility saturation.

### 2.3 The sequential explanatory expert forum

A sequential interpretive expert forum was held at the Makkah Science Forum 2025. This forum comprised 15 senior healthcare leaders, epidemiologists, and critical care specialists from the Ministry of Health and public health groups. Faculty members from the Ministry of Education (College of Medicine and College of Laboratory Medicine) were invited to participate as academic speakers, but they did not hold voting or administrative roles on the 15-member consensus committee. Over two sessions, the speakers presented operational data and digital healthcare statistics for the Hajj season. The recommendations presented in Table 2 were formulated using a simple majority consensus (over 70%). All participants provided written informed consent. The qualitative outputs were organized into thematic areas to formulate operational recommendations derived from the consensus, rather than serving as an independently validated Delphi model.

**Table 2:** Consensus framework for strengthening laboratory systems in mass gatherings: summary of strategic priorities and operational recommendations.

Operational domain	Consensus recommendations & Strategic opportunities
**Integrated triage & patient flow** *(Unified virtual routing*)	**Recommendation**: Transition from unstructured physical clinic access to standardized virtual triaging. To prevent local clinical facility saturation, future operational protocols should prioritize unified digital gateways (e.g., the Sehhaty platform) as the primary administrative and low–acuity screening filter.
	**Strategic opportunity**: Establish centralized virtual hospital networks as remote clinical escalation nodes. By managing complex but non–critical cases through remote specialty consultations, the health system can minimize the logistical strain of inter–facility patient transfers.
**Laboratory surveillance networks** ***(Centralized diagno*****stics)**	**Recommendation**: Standardize laboratory communication networks. Diagnostic outputs must be designed as dynamic, interlinked epidemiologic datasets rather than static laboratory reports. Expert consensus supports a unified workflow where field–level molecular screening data integrates directly into the Health Electronic Surveillance Network (HESN).
	**Strategic opportunity:** Integrate automated clinical alert protocols within central databases. Positive diagnostic signals from mobile or field–level PCR units should automatically notify the National Health Command and Control Center (CCC) to support pre–authorized, rapid epidemiological containment workflows, bypassing localized administrative delays.
**Molecular characterization (** ***Epidemiological monitoring*** **)**	**Recommendation:** Transition from purely culture–based phenotypic surveillance to rapid molecular characterization. Managing potential transnational biological threats and tracking antimicrobial resistance (AMR) profiles requires deploying advanced molecular diagnostic capacities closer to clinical care sites.
	**Strategic opportunity:** Integrate rapid molecular assays and pathogen characterization pathways into regional Antimicrobial Stewardship Programs (ASP). Utilizing real–time, field–deployable profiling supports data–driven empirical therapy adjustments during the mass gathering, minimizing reliance on retrospective laboratory analysis.
**Workforce & Biosafety** *(Standardization & Resilience)*	**Recommendation:** Standardize biosafety and operational clinical practices across temporary and seasonal facilities. To account for the high variability in transient, deployed healthcare staff, a unified, competency–based biosafety curriculum must be mandated for all seasonal laboratory and clinical personnel.
	**Strategic opportunity**: Implement standardized accreditation and quality–control pathways for mobile and temporary field laboratories. This ensures that high–throughput clinical capacity during peak pilgrim surges does not compromise diagnostic precision or laboratory biosafety integrity.
**Predictive analytical tools** ***(**Operational planning)*	**Recommendation**: Prioritize predictive analytical tools for logistical planning and resource allocation over clinical diagnostic automation. Computational modeling should focus on supply–chain modeling (e.g., automated reagent and clinical inventory forecasting) rather than unvalidated clinical diagnostic algorithms.
	**Strategic opportunity**: Deploy predictive spatial models of the mass gathering environment. Simulating disease transmission vectors and population movement patterns allows laboratory coordinators to pre–position testing kits, personal protective equipment, and personnel based on climatological and geographic risk projections rather than reactive demand.

This table identified by the 2025 Makkah Scientific Forum delegates. All data-driven parameters were evaluated independently of the panel's recommendation.

### 2.4 Conceptual mapping of operational and surveillance frameworks

**Figure 1:**
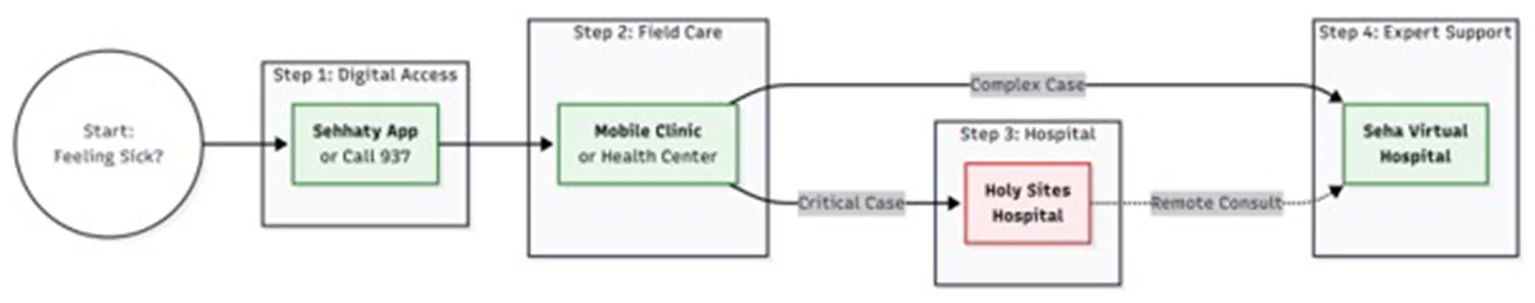
Integrated Healthcare Model implemented by the Saudi Ministry of Health (MOH) for the Hajj pilgrim care path. A flowchart reflecting the forum's endorsement of a stratified patient journey. The model proposes a strict diversion policy where digital triage and mobile clinics act as filters to protect hospital capacity. The participants highlighted the ”Remote Consult" link via the Seha Virtual Hospital as a supportive practice for expanding specialist access without physical transfer”.

To visualize the integration of quantitative operational metrics and qualitative consensus-derived recommendations, two distinct conceptual flow models were constructed to outline the health system's functional pathways:

The Triage and Patient-Flow Pathway (Figure 1): This schematic was developed by mapping healthcare utilization patterns to describe the routing of clinical inquiries through parallel virtual and physical channels. Under the health equity framework, this model illustrates how virtual triage nodes are designed to absorb low-acuity consultations, thereby preserving physical primary care and emergency department capacities for high-acuity, high-risk cohorts requiring on-site clinical stabilization.

**Figure 2:**
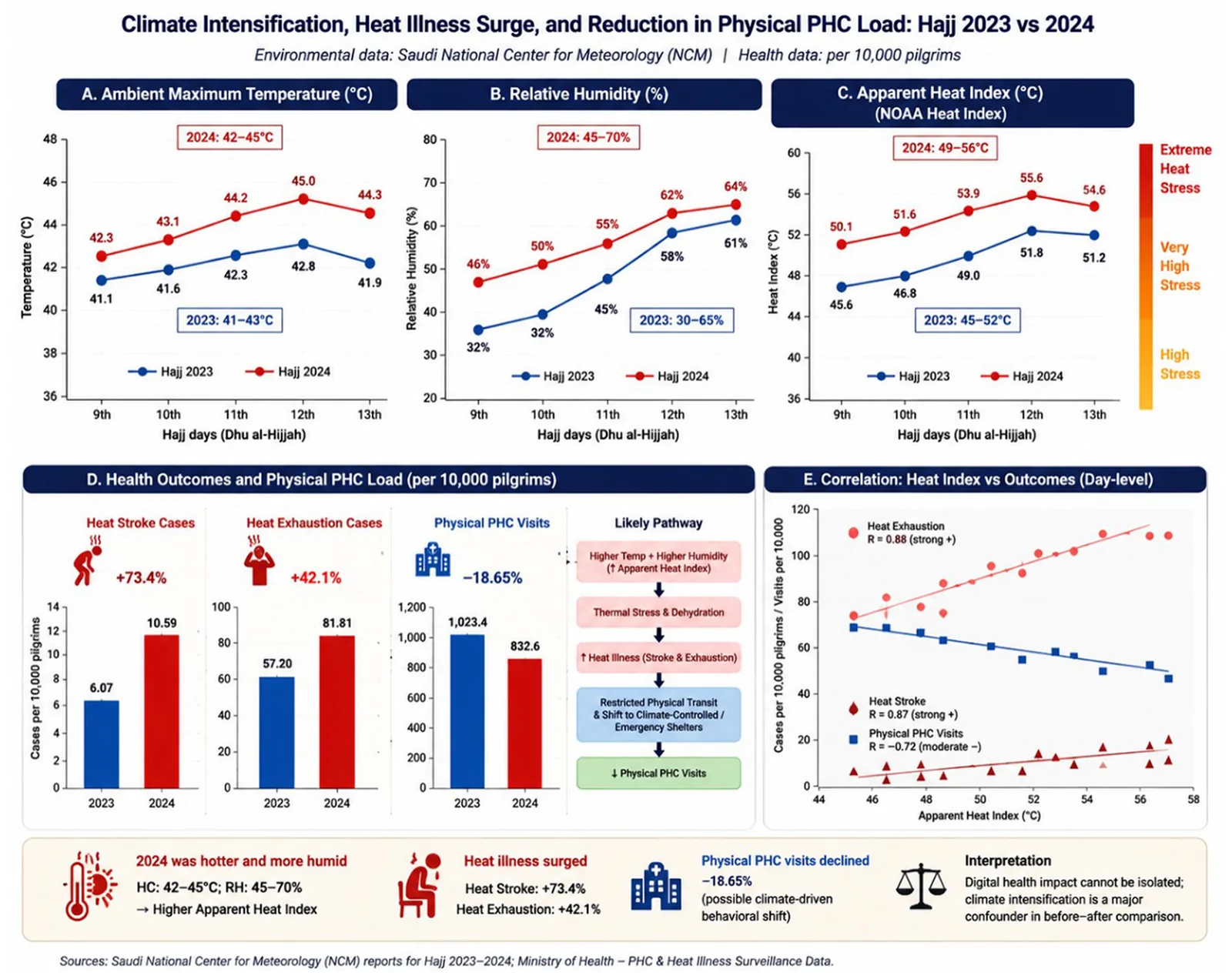
Operational Schematic of the National Public Health Surveillance and Diagnostic Laboratory Network. The flowchart illustrates the structured communication pathway connecting (1) pre-event laboratory accreditation and quality systems with (2) active molecular and biochemical testing surges. Field-level data is routed electronically to (3) the national Health Electronic Surveillance Network (HESN) database, providing diagnostic visibility to support (4) epidemiological risk assessments and operational decision-making by Weqaya (Public Health Authority) and the Command-and-Control Center (CCC).

The Surveillance and Diagnostic Framework (Figure 2): This operational flowchart was constructed by tracing data integration pathways from temporary, field-level testing units to centralized national surveillance databases. To address management risks related to reporting latency and communication silos, this model outlines a structured communication workflow designed to consolidate biochemical and molecular laboratory outputs within the Health Electronic Surveillance Network (HESN) to support epidemiological decision-making by public health authorities.

## 3 Results

Comparative operational indicators from the 2023 and 2024 Hajj seasons outline an important transition in mass gathering health coordination, focusing on the utility of virtual triage pathways to redistribute low-acuity clinical volume. Historically, health system resilience in high-density settings was evaluated primarily by physical surge capacity, such as expanding hospital bed footprints. However, the 2024 operational data suggests that managing healthcare utilization patterns is closely associated with cross-platform electronic integration and automated triage routing, rather than physical expansion alone. By establishing standardized clinical routing protocols and centralized diagnostic reporting networks, the operational response during the 2024 season indicates that mitigating communication silos is key to resolving management and coordination risks (1).

Crucially, while the implementation of virtual triage pathways co-occurred with a localized reduction in physical primary care clinic utilization, it did not protect the healthcare system against a severe surge in high-acuity presentations—such as emergency department admissions and acute heat stroke cases—driven by the extreme environmental thermal anomaly. Consequently, laboratory networks and clinical registries do not function as peripheral administrative tools; instead, they serve as the operational core of a coordinated response framework, enabling the systematic tracking of infectious and environmental hazards under substantial climatological and demographic strain (12).

### 3.1 Quantifying the crowd-to-strain ratio: primary operational load and diversion rates

The total registered pilgrim population was 1,845,223 in 2023 and 1,833,164 in 2024, representing a minor density decrease of 0.65%. Despite this stable baseline, standardized clinical utilization rates per 10,000 pilgrims shifted dramatically between the two seasons (Table 2). Physical PHC clinic visits fell by 18.11%, dropping from a rate of 1,062.06 visits per 10,000 pilgrims in 2023 (*n* = 195,974) to 869.67 per 10,000 pilgrims in 2024 (*n* = 159,425). In parallel, the integrated digital health network captured 516,585 virtual consultations during the 2024 season, representing an operational rate of 2,817.96 virtual consultations per 10,000 pilgrims and establishing an outstanding virtual-to-physical primary care utilization ratio of 3.24:1.

However, acute care utilization demonstrated a substantial upward trajectory. Standardized emergency department visits at the holy sites rose by 43.38%, increasing from 211.99 per 10,000 pilgrims in 2023 (*n* = 39,118) to 303.95 per 10,000 pilgrims in 2024 (*n* = 55,717). This clinical shift was further illustrated by the Emergency Department-to-PHC ratio, which increased from 0.200:1 in 2023 to 0.350:1 in 2024, reflecting a heavier clinical load on acute care facilities under extreme environmental conditions. Similar digital and data-driven approaches were highlighted as essential enablers of mass-gathering health security during the Fifth International Conference on Mass Gatherings Medicine in 2024, particularly in relation to real-time triage, crowd risk mitigation, and protection of critical care capacity (13–15).

**Operational Response and Digital Diversion** To neutralize the environmental surge identified in Table 2, the system aggressively activated its digital diversion nodes. Operational data confirms that the Makkah Health Cluster executed **225,296 completed virtual appointments** via the *Sehhaty* platform. This scale of virtual utilization was associated with an **18.6% reduction** in physical primary health care visits. This inverse relationship could be the “diversion hypothesis”: by successfully routing low-acuity cases away from on-site facilities during peak density, the model mitigated the “dual burden” challenge—where mass gatherings collapse local infrastructure—often cited as the primary failure point in Low- and Middle-Income Country (LMIC) settings.

Workflow Architecture (Figure 1) > The operational care pathways facilitating this primary-care triage are illustrated in Figure 1. The framework structures the clinical pathway such that initial clinical inquiries are directed through the virtual portal via the Sehhaty mobile application or the 937 tele-health service (**Step 1**). This digital screening pathway is designed to route low-acuity consultations toward mobile field clinics or virtual consultation nodes (**Step 2**), with the clinical objective of preserving physical tertiary hospital bed and emergency department capacity (**Step 3**) for high-acuity, life-threatening pathologies. Furthermore, the expert forum highlighted the utility of the ‘Remote Consultation' link, which enables field-level physicians to access clinical sub-specialty expertise via the Seha Virtual Hospital (**Step 4**). This administrative pathway supports localized clinical decision-making without requiring physical, inter-facility patient transfers, thereby minimizing transport coordination barriers in high-density sectors (16).

### 3.2 Core laboratory investigations and diagnostic capacities revision

During the 2024 season, the laboratory infrastructure executed a total of 18.88 million diagnostic investigations (~102,998 tests per 10,000 pilgrims), representing a 15.13% increase over the 16.40 million investigations executed in 2023 (~88,878 per 10,000 pilgrims). These investigations represented centralized biochemistry panels, blood counts, and molecular polymerase chain reaction (PCR) assays for infectious disease surveillance, establishing a responsive diagnostic footprint to support physical and virtual care nodes. While molecular testing was fully integrated into the epidemiologic network, high-resolution genomic sequencing and real-time variant typing were not dynamically tracked within this specific operational database and are presented here as systemic capacities rather than empirical results.

The integration of these diagnostic investigations within the broader public health response is illustrated by the operational surveillance framework shown in Figure 2. Rather than relying on isolated clinical reports, this framework describes the operational linking of standardized diagnostic outputs with centralized public health coordination channels.

This information framework describes a structured data pathway, linking pre-deployment laboratory accreditation (Phase 1) with active clinical diagnostics during the seasonal surge (Phase 2). This structure relies on Phase 3 (Data Integration), where biochemical and molecular outputs from regional and mobile laboratories are consolidated within the HESN. This electronic linkage is designed to reduce reporting latency, supporting real-time tracking of diagnostic volumes. While computational algorithms were utilized within the HESN to support logistical resource modeling, advanced genomic variant tracking and real-time evolutionary analysis of pathogen sequences were not dynamically executed on this operational dataset; instead, they represent systemic, pre-established biosecurity capacities rather than empirical findings of the current evaluation.

By coordinating laboratory diagnostic data with centralized public health surveillance channels (Phase 4), this framework provides an operational mechanism to address the data-transmission silos that historically challenge high-density environments (12, 17, 18). The integration of these temporary, event-specific diagnostic assets within the permanent national healthcare infrastructure is designed to support long-term capacity building (12, 13). These findings are consistent with the strategic health-security priorities discussed at the Fifth International Conference on Mass Gatherings Medicine, highlighting the role of coordinated laboratory and surveillance networks in managing seasonal public health demands (13).

### 3.3 Clinical heat-related illness trends and environmental burden

The 2024 Hajj season was characterized by severe environmental thermal stress, which served as a major external strain on the public health infrastructure. According to meteorological records from the Saudi National Center for Meteorology (NCM), peak ambient temperatures during the active 5-day event period (9th−13th Dhu al-Hijjah) ranged between 42 °C and 45 °C, with relative humidity peaks reaching 45% to 70%. This compound atmospheric state drove the Apparent Heat Index into extreme caution and danger thresholds, ranging from 49 °C to 56 °C.

This acute environmental shift co-occurred with a substantial increase in severe thermal pathologies. Documented cases of severe heat stroke rose by 73.36%, increasing from 1,120 cases in 2023 to 1,942 cases in 2024. Adjusted for the registered pilgrim denominators, this represents a standardized rate increase from 6.07 to 10.59 cases per 10,000 pilgrims (Table 2). Similarly, heat exhaustion cases rose by 42.10%, expanding from 10,555 cases in 2023 to 14,997 cases in 2024, corresponding to a standardized rate increase from 57.20 to 81.81 cases per 10,000 pilgrims.

To manage the operational pressure of these localized patient surges, computational algorithms and logistical resource modeling were deployed within the centralized Health Electronic Surveillance Network (HESN). Rather than relying on unvalidated clinical diagnostic software or autonomous clinical decision-making systems, these mathematical tools were restricted to supply-chain management. Specifically, they were used to forecast clinical chemistry and molecular reagent consumption rates, monitor diagnostic throughput at mobile testing units, and model sample transit logistics across regional transport bottlenecks. This targeted deployment of analytical modeling was designed to optimize laboratory inventory levels and prevent regional stockouts during the peak demand period (19).

### 3.4 Healthcare system coordination and operational redundancy

A comparative analysis of the operational datasets indicates that managing healthcare utilization patterns during extreme environmental events relies on establishing functional layers of structural and operational redundancy. Rather than dismantling physical backup systems, the electronic triage network and the physical primary care infrastructure functioned as parallel, mutually supporting tiers designed to prevent clinical saturation (Figures 1, 2).

Quantitative system metrics demonstrate that the virtual consultation portal (the Sehhaty platform and 937 tele-health service) absorbed a substantial volume of low-acuity clinical traffic. In 2024, the virtual network captured 516,585 completed virtual consultations (2,817.96 per 10,000 pilgrims), establishing a virtual-to-physical primary care utilization ratio of 3.24:1. In parallel, physical primary health care (PHC) clinic visits fell by 18.65%, dropping from 195,974 visits in 2023 to 159,425 in 2024, representing a standardized rate decline from 1,062.06 to 869.67 visits per 10,000 pilgrims.

However, this redistribution of primary care utilization did not insulate the healthcare system from high-acuity strain. Driven by the extreme environmental conditions, standardized emergency department (ED) visits at the Holy Sites hospitals rose by 42.45%, increasing from 39,118 visits in 2023 to 55,717 visits in 2024 (a rate increase from 211.99 to 303.95 visits per 10,000 pilgrims). This clinical shift is further characterized by the Emergency Department-to-PHC ratio, which rose from 0.200:1 in 2023 to 0.350:1 in 2024, indicating a heavier clinical load on acute emergency facilities.

Under these conditions of acute clinical strain, technological interoperability between the Seha Virtual Hospital, the HESN database, and localized clinical registries provided real-time visibility of healthcare utilization. This electronic coordination allowed coordinators to monitor bed occupancy and resource depletion levels continuously. In the event of local bandwidth limitations or if physical field clinics reached maximum capacity, the built-in functional redundancy allowed clinical inquiries to be dynamically routed through alternative digital or physical pathways without data fragmentation. This dual-layer framework provided the operational depth required to maintain acute-care pathways during the environmental surge (20).

### 3.5 Clinical heat-related illness trends and environmental burden

To manage the operational pressure of these localized patient surges, computational algorithms and resource modeling were integrated within the centralized Health Electronic Surveillance Network (HESN). Rather than deploying unvalidated clinical diagnostic software or autonomous clinical decision-making systems, the operational framework restricted the use of these mathematical tools to logistical and supply-chain management. Specifically, analytical models were utilized to forecast diagnostic chemistry and molecular reagent consumption rates, monitor operational throughput at high-volume mobile testing units, and model sample transit routes across regional transport bottlenecks. This targeted deployment of predictive analytics was designed to optimize laboratory inventory levels, protect diagnostic workflows, and prevent regional reagent stockouts during peak seasonal demand (19).

### 3.6 Operational interoperability and system redundancy

A comparative analysis of the operational datasets suggests that managing healthcare utilization patterns during extreme environmental surges relies on establishing functional layers of structural and operational redundancy. Rather than relying on a single triage pathway, technological interoperability was systematically integrated with intentional layers of structural backup, where the electronic triage network and the physical primary care infrastructure functioned as parallel, mutually supporting tiers designed to prevent clinical saturation (Figures 1, 2).

Quantitative system metrics indicate that the virtual care network and physical clinical facilities functioned as integrated, complementary components. In the event of localized digital bandwidth limitations or if physical field stations reached maximum clinical capacity during the thermal surge—which was characterized by a 73.36% increase in documented heat stroke cases (10.59 cases per 10,000 pilgrims)—this structural redundancy allowed clinical inquiries to be dynamically rerouted through alternative digital or physical pathways without data fragmentation.

Interoperability across platforms—specifically the electronic linkages connecting the Sehhaty portal, the HESN database, and the Seha Virtual Hospital network—provided coordinate-level real-time visibility of healthcare utilization patterns. This dual-layer operational depth was designed to redistribute low-acuity clinical volume at the primary health care (PHC) level, thereby protecting physical acute-care capacities and enabling the physical system to manage severe environmental surges without structural or electronic coordination failures (20).

## 4 Discussion

Comparative operational indicators from the 2023 and 2024 Hajj seasons outline an important transition in mass gathering health coordination, focusing on the utility of virtual triage pathways to redistribute low-acuity clinical volume. Historically, health system resilience in high-density settings was evaluated primarily by physical surge capacity, such as expanding hospital bed footprints. However, the 2024 operational data suggests that managing healthcare utilization patterns is closely associated with cross-platform electronic integration and automated triage routing, rather than physical expansion alone. By establishing standardized clinical routing protocols and centralized diagnostic reporting networks, the operational response during the 2024 season indicates that mitigating communication silos is key to resolving management and coordination risks (1).

Crucially, while the implementation of virtual triage pathways co-occurred with a localized reduction in physical primary care clinic utilization, it did not protect the healthcare system against a severe surge in high-acuity presentations—such as emergency department admissions and acute heat stroke cases—driven by the extreme environmental thermal anomaly. Consequently, laboratory networks and clinical registries do not function as peripheral administrative tools; instead, they serve as the operational core of a coordinated response framework, enabling the systematic tracking of infectious and environmental hazards under substantial climatological and demographic strain (12).

### 4.1 Integrating climate physics and confounders

Furthermore, the environmental climate profile of the 2024 season represents a major, uncontrolled confounder that complicates direct before-and-after attributions. According to the Saudi National Center for Meteorology (NCM) official reports, during the 5-day Hajj period (9th−13th Dhu al-Hijjah), the 2023 season was characterized by peak ambient maximum temperatures between 41 °C and 43 °C with relative humidity ranging between 30% and 65%. Conversely, the 2024 season experienced an intensified thermal profile, with peak ambient maximum temperatures consistently forecast between 42 °C and 45 °C and relative humidity ranging between 45% and 70%24. The day-level correlation analysis reveals that physical PHC clinic utilization was moderately negatively correlated with the Apparent Heat Index (*R* = −0.72) when the Apparent Heat Index rose to extreme.

In human biometeorology, high relative humidity combined with elevated ambient temperatures significantly elevates the apparent heat index, severely compromising the body's primary thermoregulatory mechanism of sweat evaporation. This meteorological shift explains the substantial, co-occurring surge in documented heat stroke cases (10.59 vs. 6.07 per 10,000 pilgrims; aa 73.4% volume increase) and heat exhaustion (81.81 vs. 57.20 per 10,000 pilgrims; a 42.1% volume increase). Consequently, the 18.65% decline in physical PHC visits cannot be cleanly attributed to digital health diversion alone; the elevated apparent temperature during the 2024 season may have independently restricted physical pilgrim transit or altered healthcare-seeking behaviors toward climate-controlled areas and acute emergency shelters, confounding the comparison (21).

### 4.2 The role of virtual care infrastructure: scalability and telemedical bottlenecks

The consensus identifies the “Lab-to-Action” loop within the Four-Phase Surveillance Ecosystem (Figure 2) as the core innovation of the Saudi model, directly addressing the systemic “data gaps” cited in global security reviews. While standard mass gathering protocols often rely on aggregate data that masks specific risks and fails to trigger timely responses (22, 23), the 2024 Hajj model integrated molecular outputs from field units directly into the Health Electronic Surveillance Network (HESN).

This integration was supported by non-trivial infrastructure scale: the Makkah regional laboratory network carried out **18.88 million diagnostic investigations** in 2024 (approximately 8% of the total national investigations). This massive throughput underpins the “Molecular Surge” phase, feeding the HESN with granular, real-time data rather than lagging indicators. By eliminating the communication silos identified by Tavan et al. the system ensures containment decisions are driven by live biological intelligence. This transition from passive reporting to active interrogation establishes a “predictive biosecurity” capability, neutralizing the management risks traditionally associated with delayed outbreak detection (1, 22, 30, 32).

### 4.3 Mass gatherings in the era of climate change: managing extreme environmental surges

The severe biometeorological conditions described in **Section 4.1**—where the compound effect of elevated ambient temperatures (42 °C−45 °C) and high relative humidity (45%−70%) drove the Apparent Heat Index into extreme thresholds (49 °C−56 °C)—demonstrate that health systems must adapt operational protocols to environmental stressors. To manage these pressures, the expert consensus prioritized predictive analytical modeling for logistical coordination and supply-chain management rather than autonomous clinical diagnostic systems. This conservative operational approach focused computational tools on tracking reagent inventories, monitoring diagnostic throughput, and modeling sample routing based on physical crowd movement and regional temperature patterns. This selective deployment of predictive modeling minimizes the risk of algorithmic bias within a hyper-diverse pilgrim population and ensures that digital infrastructure does not outpace local workforce capacities. Furthermore, the observed disparities in laboratory proficiency across seasonal clinical nodes underscore the critical need for standardized, rapid biosafety training to align temporary workforce capabilities with international risk assessment standards (23–25).

A primary operational finding of the 2024 season is that clinical and technological interoperability must be structurally coupled with operational redundancy to withstand extreme environmental surges. While interoperability ensures real-time clinical visibility across different healthcare tiers (e.g., matching regional laboratory diagnostics with virtual command nodes), system redundancy protects against single-point-of-failure collapses during sudden surges in heat-related illnesses. The virtual triage network functioned as a parallel redundant tier; it did not replace localized primary care clinics but served as an alternative access pathway when extreme apparent temperatures compromised physical pilgrim transit (*R* = −0.72). Future mass gathering frameworks must treat interoperability and redundancy as twin pillars: the former provides the clinical visibility to move data across platforms, while the latter provides the structural and physical depth required to maintain care-delivery pathways during major climatological events (26).

### 4.4 Strategic implications for global health security: balancing interoperability, redundancy, and policy

These operational observations align with the strategic priorities identified during the 2024 Mass Gatherings Medicine International Conference, highlighting how large-scale mass gatherings serve as critical environments for evaluating health system resilience (13, 27). Rather than viewing these events solely as acute operational liabilities, the integration of digital and physical workflows suggests that managing temporary seasonal surges can provide valuable insights for broader health system strengthening (13, 27, 33).

The electronic coordination frameworks utilized during these seasons—specifically the Seha Virtual Hospital and the Health Electronic Surveillance Network (HESN) integration—offer a reference model for managing surge-driven clinical demand. Beyond acute seasonal responses, embedding temporary diagnostic and triage assets into the permanent national biosecurity infrastructure supports long-term capacity building (2, 28). This integration aligns with international guidelines on health system strengthening, indicating that temporary surge demands can be leveraged to address chronic operational gaps, ultimately leaving a functional public health infrastructure that benefits host communities long after the event concludes (1, 34). Importantly, because this framework relies on pre-existing digital maturity, its transferability to mass gatherings in low-to-middle-income countries (LMICs) requires careful adaptation to local infrastructure capacities.

## 5 Limitations

Evaluating the shifts in primary care utilization patterns associated with the virtual triage rollout requires acknowledging three key methodological constraints. **First**, the observed operational outcomes are highly dependent on the pre-existing digital maturity and centralized health information infrastructure of the host country. Consequently, the direct transferability of this integrated telemedical model to mass gatherings in low-to-middle-income countries (LMICs)—which may lack a highly integrated national digital backbone to coordinate seasonal clinical surges—remains to be empirically evaluated in resource-constrained environments rather than assumed.

**Second**, because our analysis relied on high-level, aggregate operational metrics, we face a substantial analytical limitation regarding demographic selection bias and the digital divide. The retrospective, administrative dataset lacks patient-level clinical linkage and socioeconomic, linguistic, or age-related stratification. It is highly probable that younger, technologically literate, and bilingual (Arabic- or English-speaking) pilgrims utilized the Sehhaty virtual platform at disproportionately higher rates. Conversely, old adults, non-digitally literate, or linguistically isolated cohorts may have faced substantial barriers to accessing virtual triage, potentially forcing continued reliance on physical clinical networks. Because aggregate data cannot track individual patient-level pathways, we cannot definitively determine whether vulnerable pilgrim populations were successfully served by virtual nodes or disproportionately excluded, presenting a vital area for future equity-focused research (31).

**Third**, this study evaluates two consecutive Hajj seasons, presenting a short-term operational snapshot rather than a multi-year longitudinal assessment. While this comparative analysis highlights immediate operational shifts under seasonal surge conditions, the inter-seasonal durability and long-term capacity-building impact of these virtual care assets on the permanent host health infrastructure remain unquantified. Addressing these epidemiological gaps requires future prospective, multi-cycle investigations that track clinical utilization cohorts across multiple successive mass gathering events and diverse geopolitical settings.

**Figure 3:**
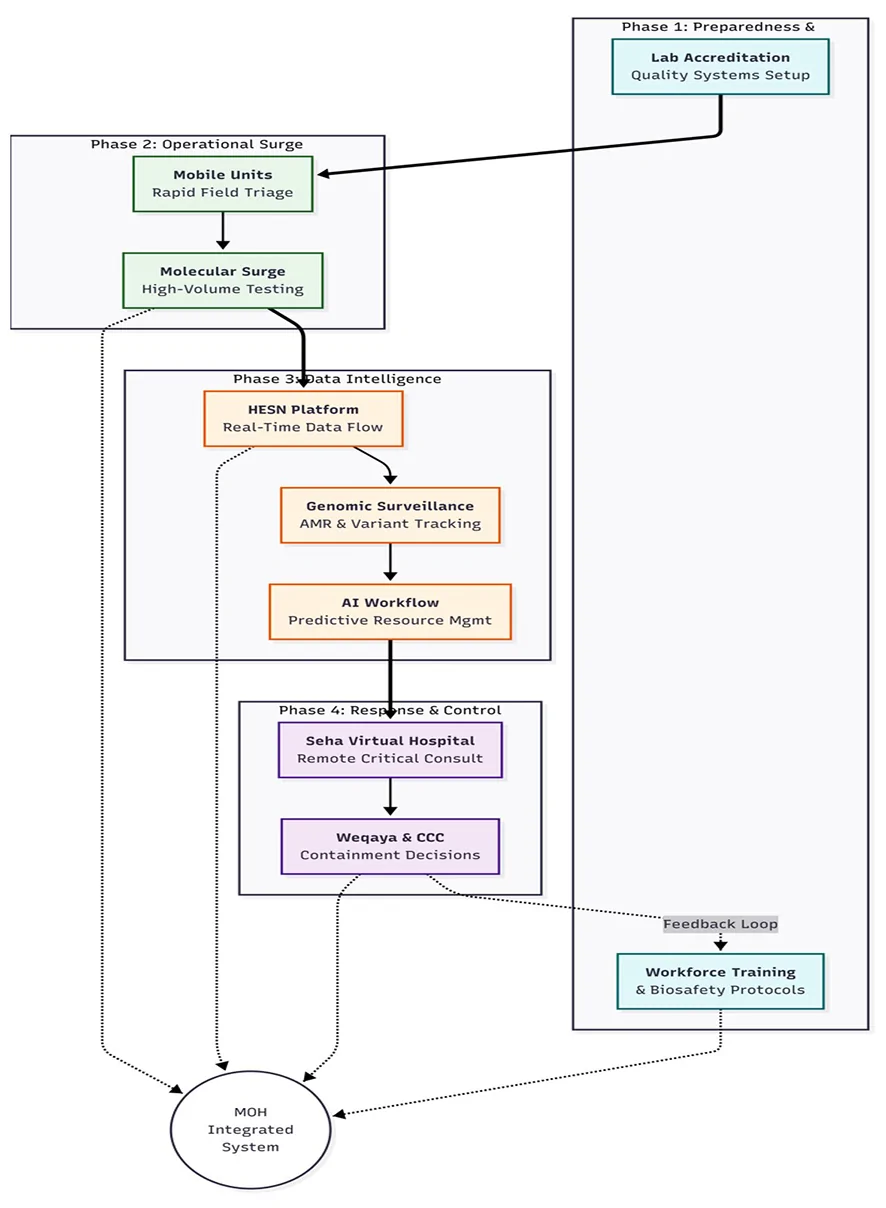
Operational Schematic of the Digital-First Triage and Diversion Model during the 2023–2024 Hajj Mass Gatherings. The flowchart illustrates the structured integration of digital triage channels (left), virtual clinical command nodes (center), and physical healthcare facilities (right), underpinned by a centralized diagnostic laboratory and epidemiological surveillance core (bottom). Under the extreme environmental thermal pressures of the 2024 season—characterized by peak ambient temperatures of 42 °C to 45 °C and relative humidity peaks of 45 % to 70% the model serves as a vital clinical buffer. By successfully absorbing 516,585 virtual consultations (representing a virtual-to-physical PHC ratio of 3.24:1), the virtual nodes mitigated the low-acuity burden on physical primary health care (PHC) clinics (which saw an 18.65 % decline in visits). This localized primary care offload preserved physical emergency department (ED) capacity and acute hospital beds, enabling the physical system to absorb the 73.36 % surge in severe, documented heat stroke cases (*n* = 1,942) and the 42.45 % increase in emergency department visits (*n* = 55,717). Google Gemini -Flash-3.5- was utilized to generate the chart from raw data. *The apparent heat index is what the temperature feels like to the human body when relative humidity is combined with the air temperature.

**Fourth**, because this is an observational, retrospective study, we cannot establish definitive causality. The observed associations between the digital care rollout and the decline in physical PHC utilization are ecological, lacking individual-level clinical linkage. We cannot separate true clinical substitution from induced telemedical demand, as aggregate-level data cannot track whether virtual users would have otherwise presented to physical clinics. Furthermore, the observed decrease in physical PHC visits is heavily confounded by the 2024 environmental thermal anomaly. As mapped in Figure 3, the moderate negative correlation between the Apparent Heat Index and physical clinic visits (*R* = −0.72) suggests that extreme apparent temperatures may have independently restricted physical pilgrim transit or shifted care-seeking behaviors toward climate-controlled areas, independently confounding the before-and-after comparison.

## 6 Conclusion

The comparative operational evaluation of the 2023 and 2024 Hajj seasons demonstrates that the integration of virtual healthcare platforms was associated with a substantial, localized redistribution of primary care utilization during a high-density mass gathering. While overall pilgrim density remained relatively stable, the deployment of a unified virtual consultation system co-occurred with an 18.65% reduction in physical primary health care clinic visits, representing a virtual-to-physical primary care utilization ratio of 3.24:1. This finding indicates that virtual networks can function as effective operational buffers to absorb low-acuity clinical volume.

However, these findings also demonstrate that digital primary care triage does not decouple health system strain in acute, life-threatening clinical categories during extreme environmental events. Under the biometeorological pressures of the 2024 season—where elevated ambient temperatures (42 °C−45 °C) and increased relative humidity (45%−70%) severely compromised physiological heat-dissipation mechanisms—acute thermal pathologies and emergency department visits surged by 73.36% and 42.45%, respectively. This underscores that while virtual care networks can successfully mitigate low-acuity clinical overcrowding, physical emergency facilities and specialized field stabilization units remain indispensable for managing critical, climate-driven surges.

Ultimately, these results suggest that the strategic utility of digital health platforms lies in their role as clinical buffers rather than absolute system-wide protective barriers. To optimize health system resilience during large-scale gatherings, the global public health community must transition from localized, *ad-hoc* emergency responses to standardized, digitally integrated care architectures that support physical clinical infrastructures under extreme environmental conditions (22).

## Data Availability

The raw data supporting the conclusions of this article will be made available by the authors, without undue reservation.

## References

[1] Tavan A Tafti AD Nekoie-Moghadam M Ehrampoush M Vafaei Nasab MR Tavangar H . Risks threatening the health of people participating in mass gatherings: a systematic review. J Educ Health Promot. (2019) 8:209. doi: 10.4103/jehp.jehp_214_19 31807599 PMC6852309

[2] Alshahrani NZ Al Awaidy S Koul P Alqahtani JS Memish ZA Zumla A . The health equity imperative in mass gathering medicine: a narrative review of global challenges and strategies. Mass Gather Med. (2025) 4:100043. doi: 10.1016/j.mgmed.2025.100043

[3] Azhar LE Almalki FY Aljadani HM Elhassen IM Fouz N Memish ZA . Epidemiology of Infectious Diseases in Mass Gatherings: Challenges and Opportunities. Mass Gather Med. (2025) 4:100036. doi: 10.1016/j.mgmed.2025.100036

[4] Tran KT Nguyen KD Nguyen TN Pham LT Nguyen LM Nguyen PH . The role, challenges, and solutions of laboratories in disaster medicine: a systematic review. Front Public Health. (2025) 13:1726280. doi: 10.3389/fpubh.2025.1726280 41607900 PMC12834775

[5] World Health Organization: Strengthening public health readiness for mass gatherings in the Eastern Mediterranean Region. Cairo: WHO Regional Office for the Eastern Mediterranean (2023).

[6] Yezli S Alotaibi BM. Mass gatherings and mass gatherings health. Saudi Med J. (2016) 37:729. doi: 10.15537/smj.2016.7.15419 27381530 PMC5018634

[7] Arbon P. Mass-gathering medicine: a review of the evidence and future directions for research. Prehosp Disaster Med. (2007) 22:131–5. doi: 10.1017/S1049023X00004507 17591185

[8] Hoang V-T Gautret P. Infectious diseases and mass gatherings. Curr Infect Dis Rep. (2018) 20:44. doi: 10.1007/s11908-018-0650-9 30155747 PMC7088693

[9] Arbaein TJ Alharbi KK Alfahmi AA Alharthi KO Monshi SS Alzahrani AM . Makkah healthcare cluster response, challenges, and interventions during COVID-19 pandemic: a qualitative study. J Infect Public Health. (2024) 17:975–85. doi: 10.1016/j.jiph.2024.04.007 38631067

[10] Benis A Haghi M Tamburis O Darmoni SJ Grosjean J Deserno TM. Digital emergency management for a complex one health landscape: the need for standardization, integration, and interoperability. Yearb Med Inform. (2023) 32:027–35. doi: 10.1055/s-0043-1768742 38147847 PMC10751113

[11] Blanchet K Nam SL Ramalingam B Pozo-Martin F. Governance and capacity to manage resilience of health systems. Health Policy Plan. (2017) 32:431–9. doi: 10.15171/ijhpm.2017.36 28812842 PMC5553211

[12] Weqaya. Antimicrobial resistance action plan: Kingdom of Saudi Arabia 2022–2025. Riyadh: Public Health Authority (2022).

[13] Khan A Alahmari A Arbon P Wang N Jokhdar H. Legacy in global health security: insights from the 5th international conference for mass gatherings medicine. J Infect Public Health. (2024) 17:1–3. doi: 10.1016/j.jiph.2023.12.029 38272756

[14] Comfort LK Boin A Demchak CC. Designing resilience: Preparing for extreme events. Pittsburgh (PA): University of Pittsburgh Press (2010). doi: 10.2307/j.ctt5hjq0c

[15] Boin A McConnell A. Preparing for critical infrastructure breakdowns: the limits of crisis management and the need for resilience. J Contingencies Crisis Manag. (2007) 15:50–9. doi: 10.1111/j.1468-5973.2007.00504.x

[16] Al Aseri Z Côté M Baharoon S Alabdulaali MK Altamimi S Arntfield R. AI in critical care: A narrative review of prospective applications and future potential in KSA's health transformation 2030. J Taibah Univ Med Sci. (2025) 20:359–64. doi: 10.1016/j.jtumed.2025.05.007 40548347 PMC12182355

[17] Kumar R Singh A Kassar ASA Humaida MI Joshi S Sharma M. Adoption challenges to artificial intelligence literacy in public healthcare: an evidence based study in Saudi Arabia. Front Public Health. (2025) 13:1558772. doi: 10.3389/fpubh.2025.1558772 40371275 PMC12076014

[18] Dobrow MJ Bytautas JP Tharmalingam S Hagens S. Interoperable Electronic Health Records and Health Information Exchanges: Systematic Review. JMIR Med Inform. (2019) 7:e12607. doi: 10.2196/12607 31172961 PMC6592487

[19] Memish ZA Steffen R White P Dar O Azhar EI Sharma A . Mass gatherings medicine: public health issues arising from mass gathering religious and sporting events. Lancet. (2019) 393:2073–84. doi: 10.1016/S0140-6736(19)30501-X 31106753 PMC7159069

[20] Gellert GA Rasławska-Socha J Marcjasz N Price T Heyduk A Mlodawska A . The role of virtual triage in improving clinician experience and satisfaction: a narrative review. Telemed Rep. (2023) 4:tmr. 2023.0020. doi: 10.1089/tmr.2023.0020 37529770 PMC10389257

[21] National Center of Meteorology. Documentation Library. Hajj Reports, Hajj 2024 (NCM Report 1445 H, Report No. 8. Available online at: https://www.ncm.gov.sa/Ar/MediaCenter/Reports/Pages/HajjSeason.aspx (Accessed June 20, 2026).

[22] Alasiri AA Mohammed V. Healthcare transformation in Saudi Arabia: an overview since the launch of vision 2030. Health serv insights. (2022) 15:11786329221121214. doi: 10.1177/11786329221121214 36081830 PMC9445529

[23] Ganser I Thiébaut R Buckeridge DL. Global Variations in Event-Based Surveillance for Disease Outbreak Detection: Time Series Analysis. JMIR Public Health Surveill. (2022) 8:e36211. doi: 10.2196/36211 36315218 PMC9664335

[24] World Health Organization: Health–Security Interface Technical Advisory Group: 2025 annual meeting summary report= Groupe consultatif technique sur l'interface santé-sécurité: compte rendu de la réunion annuelle de 2025. Wkly epidemiol rec. (2025) 100:335-341.

[25] Speizer S Raymond C Ivanovich C Horton RM. Concentrated and intensifying humid heat extremes in the IPCC AR6 regions. Geophys Res Lett. (2022) 49:e2021GL097261. doi: 10.1029/2021GL097261

[26] Kruk ME Gage AD Arsenault C Jordan K Leslie HH Roder-DeWan S . High-quality health systems in the Sustainable Development Goals era: time for a revolution. Lancet Glob Health. (2018) 6:e1196–252. doi: 10.1016/S2214-109X(18)30386-3 30196093 PMC7734391

[27] Abougazia F Saleh BM Chun S. Event-Based Surveillance in mass gatherings: a global scoping review on effectiveness, scope, and lessons learned. Front Public Health. (2026) 14:1762219. doi: 10.3389/fpubh.2026.1762219 41799468 PMC12961754

[28] Alharbi KK Alfaifi MS Alzahrani AM Alkathiri AS Sinky TH. Disease Burden and Pattern of Healthcare Utilization Among Pilgrims During Hajj 2024: A Cross-Sectional Analysis. Ann Glob Health. (2026) 92:25. doi: 10.5334/aogh.4956 41835210 PMC12985899

[29] Ministry of Health. Statistical Yearbook 1445H.(2014). Available online at: https://www.moh.gov.sa/en/Ministry/Statistics/book/Pages/default.aspx (Accessed April 15, 2026).

[30] Chen L Meng QH. Advancing laboratory diagnostics for future pandemics: challenges and innovations. Pathogens. (2025) 14:1135. doi: 10.3390/pathogens14111135 41305372 PMC12655715

[31] Roberts ET Mehrotra A. Assessment of disparities in digital access among Medicare beneficiaries and implications for telemedicine. JAMA Intern Med. (2020) 180:1386–9. doi: 10.1001/jamainternmed.2020.2666 32744601 PMC7400206

[32] Lubin IM Astles JR Shahangian S Madison B Parry R Schmidt RL . Bringing the clinical laboratory into the strategy to advance diagnostic excellence. Diagnosis. (2021) 8:281–94. doi: 10.1515/dx-2020-0119 33554526 PMC8255320

[33] Al-Tawfiq JA Memish ZA. Mass gathering medicine: 2014 Hajj and Umra preparation as a leading example. Int J Infect Dis. (2014) 27:26–31. doi: 10.1016/j.ijid.2014.07.001 25128639 PMC7110515

[34] World Health Organization. WHO Global strategy on digital health 2020-2024. Geneva: World Health Organization (2023).

